# Intense Short-Video-Based Social Media Use reduces the P300 Event-Related Potential Component in a Visual Oddball Experiment: A Sign for Reduced Attention

**DOI:** 10.3390/life14030290

**Published:** 2024-02-22

**Authors:** Peter Walla, Yu Zheng

**Affiliations:** 1 Freud CanBeLab (Cognitive & Affective Neuroscience and Behavior Laboratory), Faculty of Psychology, Sigmund Freud University, Freudplatz 1, 1020 Vienna, Austria; 2 Faculty of Medicine, Sigmund Freud University, Freudplatz 3, 1020 Vienna, Austria; 3 School of Psychology, Newcastle University, University Drive, Callaghan, NSW 2308, Australia

**Keywords:** electroencephalography (EEG), brain imaging, event-related potential (ERP), social media, short videos, oddball paradigm, attention, concentration, social media overuse, non-conscious processing

## Abstract

The birth and following growth of social media platforms has influenced a lot. In addition to beneficial features, it has long-been noticed that heavy consumption of social media can have negative effects beyond a simple lack of time for other things. Of particular interest is the idea that consuming short videos lasting only fractions of a minute and watched one after another can lead to deficits in concentration and attention. Completing the existing literature that already reports evidence for attention deficits related to heavy social media use, the present study aims to contribute to this acute topic by adding neurophysiological data to it. In particular, this study made use of a well-known experimental paradigm, which is able to detect attention-related changes on a neurophysiological level. The so-called oddball paradigm was applied and the hypothesis that heavy social media users mainly consuming short videos show a reduced P300 event-related potential (ERP) component was tested, which has been found to reflect attention-related brain functions. For this, we invited twenty-nine participants and designed a visual oddball experiment including a white circle on black background as the low-frequency target stimulus and a white triangle on black background as the high-frequency non-target stimulus. On the basis of their self-reported short-video-based social media usage habits, all participants were grouped into heavy (more than 4 h daily usage) and regular (below 3 h daily usage) users, and finally data from 14 heavy and 15 regular users were further analyzed. It was found that only regular users show a clear P300 ERP component, while this particular brain potential amplitude reflecting attentional processes was significantly reduced in heavy users. This result provides empirical brain imaging evidence that heavy short-video-based social media use indeed affects attentional brain processes in a negative way.

## 1. Introduction

Instant messaging and various forms of other social media have advantages as well as disadvantages. While the advantages seem quite obvious, it is the disadvantages that need specific attention and also research in order to highlight respective problems. In a very recent study, unilateral fake friendship (with digital celebrities) was investigated via recording brain potential changes that were later calculated into event-related potentials (ERPs) [1]. In this case, the outcome was actually positive when it came to the question of if social media consumption has negative influences on human beings. The respective authors compared ERPs that were elicited by names of followed and adored influencers and other digital celebrities, ERPs that were elicited by names of real-life loved friends and relatives, and ERPs that were elicited by names without any affiliation to the study participants. Most importantly, they found that even though unilateral fake friendship is often perceived as an intense and real friendship, the brain responded significantly differently to fake versus real friends. Such studies show that social media use might not be as harmful as often thought. However, of course there is a long list of negative social media effects of various kinds. For instance, maladaptive social media use can lead to behavioral addiction symptoms [2,3,4]. Montag and Walla [5] highlight the numerous problems of digital overuse and emphasize why we all suffer from it.

But, social media have many different faces, some of which might be more problematic than others. Since more and more platforms have arose that offer the ability to both produce and consume short video sequences, one of the most important conscious functions, namely attention, has made it into the focus of interest. Attention is so basic and yet so crucial to many highly complex tasks. Via attentional processes, the brain is able to filter all incoming information, focusing on important and ignoring unimportant information. Attention supports higher-level cognition including decision making, planning, and general reasoning [6]. Vedechkina and Borgonovi [7] report that research demonstrating a negative association between technology and attention is thus often used to assert the negative impact of technology (in particular social media) on higher-level cognitive functions, like working memory, executive control, and learning.

Especially, repetitive attention shifts in addition to multitasking during intense technology use, including the consumption of short-video-based social media, have been discussed as causing attention-related problems [8]. Constant content changes, often occurring after only fractions of a minute, divide attention at the cost of sustained attention [9]. Crucially, a recent systematic review on social media and their negative aspects included a strong emphasis on the fact that most research is still based on self-report [10]. Self-report-based studies introduce problems of social desirability bias [11], among others. In addition to that, survey questions about preferences, likes and dislikes, or similar constructs involve affective information processing that is in fact difficult to verbalize [12]. This phenomenon has been labeled “cognitive pollution” [13,14]. Potential bias together with cognitive pollution related to self-report have made us think about another approach to contributing a better understanding of social media impact. A good solution to this problem is the use of objective measurements, in particular brain imaging tools (also called neuroimaging tools) that allow for one to watch the brain at work. A recent review highlights the importance of neuroscience in understanding the influence of media on adolescents’ brains [15].

In response to that, we decided to use a well-known brain potential component (i.e., a distinct brain activity) that has been found to reflect attentional processes in the brain, the so-called P300 component, to investigate short-video-based social media effects on attention [16]. It is the positive potential signal resulting from calculating the ERPs collected in the frame of a so-called oddball paradigm that allows us to objectively measure general cognitive and attention-related functioning in the human brain via utilizing electroencephalography (EEG) [16]. In summary, during a series of stimuli presentations (usually tones or images), there is one non-target stimulus that appears more often than a target stimulus that appears more rarely. In other words, the simplest version of an oddball experiment contains only two different stimuli that are presented one after each other in random sequence with one of the two appearing with a lower frequency. While study participants are asked to pay attention and distinguish (per button press) between target and non-target stimuli, their brain potential changes are recorded via EEG. Often replicated, the very robust finding has always been that the rare stimuli elicited a more positive averaged brain potential amplitude (event-related potential, ERP) than the more frequent stimuli at around 300 ms after stimulus onset.

The goal of this study was to design an oddball experiment with visual stimuli and to invite participants to the FreudCanBelab (Freud Cognitive & Affective Neuroscience and Behavior Laboratory) at Sigmund Freud University in Vienna, where their brain activities would be recorded with electroencephalography (EEG) during their task to distinguish between two simple objects, a circle and a triangle. Afterwards, brain potentials were calculated into ERPs, and ERPs resulting from the rare stimuli were compared to ERPs resulting from the more frequent stimuli. The objective of this study was to test the hypothesis that heavy short-video-based social media users show a reduced P300 ERP component, which then would be interpretated as a sign of weaker attention-related brain processes when compared to regular users. The rationale for this is based on the literature highlighting the connection between attention as a brain function and the P300 ERP component.

## 2. Materials and Methods

### 2.1. Participants

**Figure 1:**
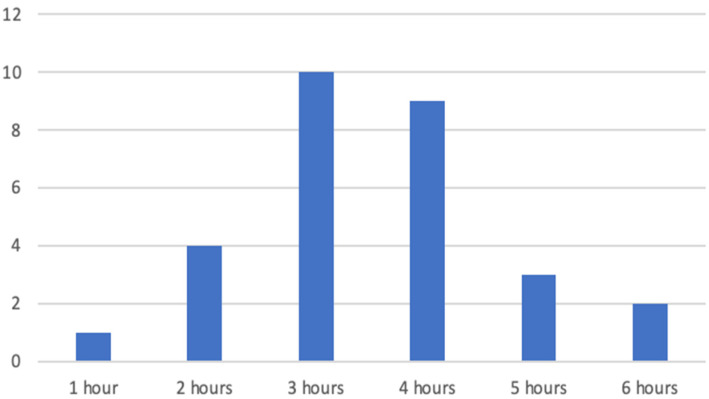
The *y*-axis shows the number of participants that self-reported respective hours of social media use per day. Finally, the heavy-user group included all participants reporting social media use of at least 4 h a day. The regular-user group included all participants reporting a daily social media use of maximally 3 h per day.

Through convenience sampling, 29 participants were recruited to take part in this study. All of them were asked how much time they usually (general use) spend on short-video-based social media per day. The options were “less than 30 min”, “about an hour”, “about 2 h”, “about 3 h”, “about 4 h”, “about 5 h”, and “more than 5 h”. Finally, 14 participants reporting average short-video-based social media usage above 4 h were grouped as heavy users (one male subject), while 15 participants reporting their respective average usage to be below 3 h were grouped as low users (two males) (Figure 1 shows the distribution of hours of use across all participants). The mean age of the regular users was 21.87 years (SD = 1.88), and the mean age of the heavy users was 21.07 years (SD = 1.21). As collected via a demographics survey, all participants had no neuropathological history, they had normal or corrected to normal vision and were all right-handed. None of them were on any medication or using drugs.

### 2.2. Stimuli

**Figure 2:**
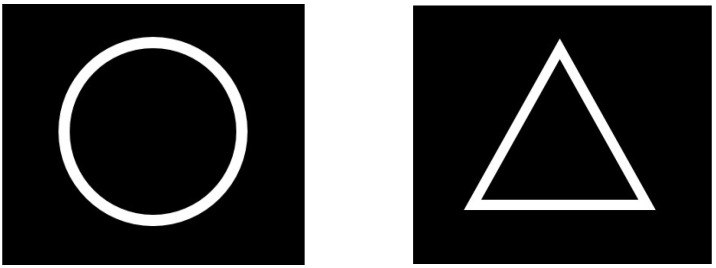
Both visual stimuli used for the oddball paradigm: (**Left**) the white circle on black background, which was used as the rare (target) stimulus, is shown. (**Right**) the white triangle on black background, which was used as the more frequent (non-target) stimulus, is shown.

A visual oddball paradigm contains two different stimuli appearing at different presentation rates, one is presented more often than the other. In this study, a white circle on black background was used as the rare stimulus (target stimulus) and a white triangle on black background was used as the more frequent stimulus (non-target stimulus) (see Figure 2). The more frequent stimulus appeared 4-times more often than the rare stimulus.

### 2.3. Procedure

Once having arrived at the lab, the participants were introduced to the purpose of this study. If they agreed to participate, they signed an informed consent form and filled in a short demographics survey. Then, a so-called actiCAP from Brain Products with 64 electrodes embedded was applied to their heads and connected to the amplifier. They were instructed to sit still and blink with their eyes only when they saw a fixation cross and to avoid blinking during visual object presentations. Each object presentation lasted only 300 ms (white object on a black background) on a computer monitor placed on a table in front of the participants, who sat on a comfortable chair. This was followed by a blank black screen for 1 s and a white fixation cross on a black background for 1 s, with a final blank black screen again for 1 s until the next object was presented. The eye-to-screen distance was about 1 m, and all visual stimuli were presented so as to stimulate foveal receptors only (no peripheral field stimulation). The participants were instructed to indicate via a button press whether they saw a circle or a triangle.

### 2.4. Electroencephalography (EEG)

Brain potential changes were recorded with a 64-channel actiCHamp Plus System from Brain Products. This system had active electrodes embedded in an actiCAP connected to an amplifier, which was operated by a powerful lithium-ion battery pack. The distribution of all 64 electrodes followed the well-known 10–20 system. The locations of electrodes on the cap were fixed, which resulted in very stable electrode positions. Brain potentials were sampled with a rate of 1 kHz (filtered: DC to 100 Hz). Impedance was kept equal to or below 10 kΩ, and electrode location Cz was used as reference. A midfrontal electrode position on the forehead was used as the ground electrode. Offline, all EEG data were down-sampled to 250 Hz, and a bandpass filter from 0.1 to 30 Hz was applied in preparation for the following EEG data processing (see [1]). A usual stimulus-elicited ERP shows deflections of maximally 10 Hz; as such, a bandpass filter thus smoothed the data without losing the signal.

### 2.5. Analysis

EEG data processing was performed by using an updated version (6.4.9) of the initial EEGDISPLAY 6.1.5 software (not commercially available), which was developed by Fulham [17]. Epochs from 100 ms before stimulus onset (baseline) until 1 s after stimulus onset were generated. All epochs contaminated by visible artifacts were manually selected and excluded, and those with an electrooculogram (EOG) amplitude exceeding ±75 mV were automatically excluded (same procedure as in the Walla et al. study [1]).

**Figure 3:**
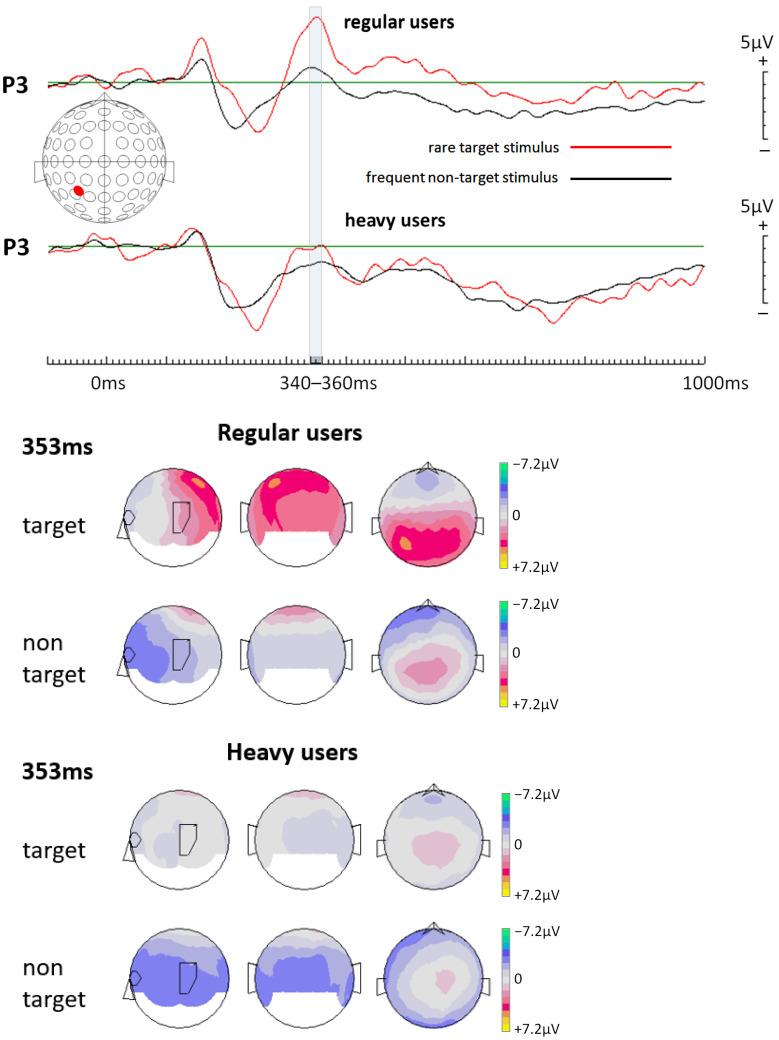
(**Top**) Event-related potentials (ERPs) generated for electrode P3 (left parietal) (positivity is up). The time window from 340 ms to 360 ms after stimulus onset (marked with a grey bar) revealed a significant group effect with respect to ERP differences between the target and non-target stimulus conditions. Note that regular users show a more pronounced difference between these two conditions compared to heavy users. Clearly, heavy social media users show a significantly reduced P300 ERP component compared to regular users, which is interpreted as reflecting lower attention capacities in heavy users. (**Bottom**) Topographical maps created for a single time point (353 ms) within the time window showing a significant effect that rare target stimuli elicit a clear P300 ERP component only in regular social media users.

**Figure 4:**
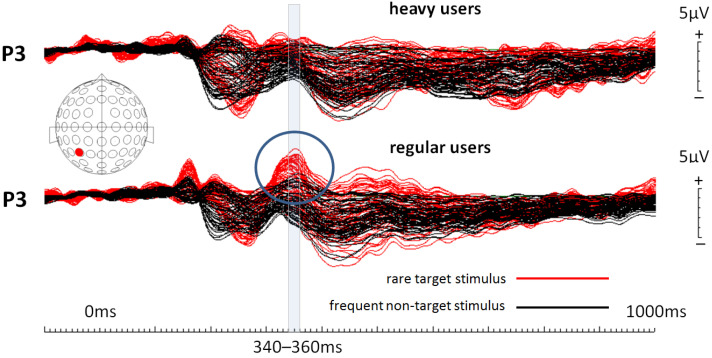
Butterfly plots for both participant groups (positivity is up). Note that heavy social media users clearly show a much-reduced P300 ERP component even when overlaying ERPs from all electrode locations. The blue circle marks the P300 ERP component in regular users.

Event-related potentials (ERPs) were calculated for each of the two object conditions for each participant and, finally, grand averages were calculated for each condition across all participants for both groups separately, including all 64 electrodes. Visual inspection of the overlaid ERPs at all 64 electrodes as well as a literature search on the P300 ERP component in the frame of an oddball paradigm [18] resulted in the decision to focus the following statistical analysis only on data collected at electrode location P3 (left parietal). This electrode location very-well represents P300 amplitude modifications. Respective ERPs from this location are shown in Figure 3, as well as topographical maps for both regular and heavy users. For statistical analysis, EEG data from all participants were further down-sampled, resulting in data points averaged across 20 ms time windows spanning from 200 ms to 520 ms after stimulus onset. Crucially, this time window included potential values before and after 300 ms post-stimulus onset to ensure the capturing of P300 amplitude changes. Visual inspection of the calculated ERPs confirms the choice of this time window. With those data points (amplitude values), an ANOVA (analysis of variance; repeated measures; Greenhouse–Geisser corrected) was calculated for every single 20 ms time window following a 2 × 2 experimental design. The first factor “condition” had the two within-subject levels, “target” and “non-target”. The second factor “group” had the two between-subject levels, “regular users” and “heavy users”. Finally, butterfly plots were generated for both groups (regular and heavy users) to illustrate the P300 effect across all electrodes (Figure 4).

## 3. Results

### 3.1. Event-Related Potentials (ERPs) and Topographical Maps

Visual inspection of the calculated ERPs for electrode location P3 shows very clearly that only in regular, but not in heavy users, can one see a well-pronounced P300 ERP component (Figure 3, top). As outlined later under 3.2., analytical statistics underlies this finding most significantly for the time window from 340 ms to 360 ms after stimulus onset. Topographical maps taking all electrode locations into account also highlight a well-pronounced P300 component spread over parietal locations, but with a maximum positivity peak at location P3 for a distinct time point at 353 ms after stimulus onset (Figure 3, bottom).

### 3.2. Analytical Statistics

ANOVAs of the 20 ms time intervals from 200 ms to 520 ms after stimulus onset (see Table 1) resulted in two significant condition * group interactions, with the lowest *p*-value for the interval from 340 ms to 360 ms. This time window is indicated in Figure 3 and Figure 4. In this time window, only regular users showed a well-pronounced P300 ERP component.

## 4. Discussion

The research fields of social media usage and its potential effects on attention in particular are still in their early stages. However, it is clear that there are generally both positive and negative effects in response to social media use [19,20,21,22]. Small et al. (2020) [23] have recently reported that some aspects of digital technology, such as distinct video games or online tools, can activate neural circuits in a positive way. However, such benefits require a certain limit regarding screen time. On the other hand, extensive screen time (several hours each day) can lead to various harmful effects among which is reduced attention or even attention-deficit hyperactivity disorder (ADHD). Even though a causal relation between high-frequency use and increased ADHD still needs to be confirmed (Ra et al., 2018) [24], there is reason to believe that at least reduced attention is indeed a likely consequence of high-frequency social media use. This idea, or rather assumption, formed the basis for the present study, which went even further by narrowing social media use down to mainly consuming short-video-based digital content. In most cases, video-based social media means several dynamic content changes per minute, which use up attention resources that in the long run might suffer from oversaturated functioning. In such cases, dived attention is busily following constantly changing content at the cost of sustained attention.

Most of the prior research on this or on similar topics has been performed by demanding explicit responses from study participants, and Orben (2020) [25] has pointed out the domination of a rather low-quality science standard. Due to potential cognitive pollution (see Walla et al., 2011; Walla and Panksepp 2013) [13,26], and since the advent of objective technology has allowed one to watch the brain at work, we decided to introduce a new research approach to the field of effects of social media on human subjects. We followed a paradigm shift from survey-based studies to measuring brain activities. In line with this notion, the present study using EEG to record brain potential changes (i.e., neural activity) might mark a new direction for investigating the possible harmful effects of social media use. EEG brings with it a quite elegant way to objectively measure attentional processes in human brains by applying a so-called oddball paradigm that has been repeatedly shown to lead to brain responses reflecting attention-related brain functions, which can be detected by an EEG system after generating so-called ERPs (event-related potentials). Thereby, a well-pronounced positive ERP amplitude, the so-called P300 component, appears in the signal at around 300 ms after the presentation of a rare stimulus. This is not the case after presentations of a more-often-appearing stimulus.

The P300 ERP component has been intensively investigated since its first detection. It becomes more pronounced as soon as brain processes related to selective attention are involved (Donchin and Coles 1988; Johnson 1988) [27,28]. In other words, the magnitude of its amplitude varies as a function of the attentional resources involved in dealing with a presented stimulus (Johnson 1998). It seems that anything that grabs attention increases the positive ERP amplitude at or around 300 ms after stimulus onset. For instance, Gray et al. (2004) [29] have shown that autobiographical self-relevant stimuli like one’s own name increases it. The most common means of triggering a clear P300 ERP component is to use any version of the abovementioned oddball paradigm, during which two different stimuli are randomly presented one after each other with one of the two stimuli appearing more often than the other. The result is a larger calculated P300 ERP component elicited by the less-often-appearing stimulus category (Donchin 1981) [30].

In the present study, we applied a very simple version of a visual oddball paradigm and by doing so we found a well-pronounced P300 ERP component in the participant group comprised of regular users (social media usage with a focus on short videos below 3 h each day). On the other hand, and most interestingly, the participants that reported as heavy social media users with a focus on short videos (more than 4 h each day) showed a much smaller (significantly smaller) P300 ERP component, indicating less attention-related brain activity in this group.

By using similar oddball paradigms, reduced P300 ERP components have been found in various psychological disorders such as depression [31] (Klawohn et al., 2019), schizophrenia [32,33] (Kawasaki et al., 1997; Vianin et al., 2002), and ADHD (attention-deficit/hyperactivity disorder) [34] (Kallen et al., 2020). Hünerli et al. [35] (2019) found decreased P300 amplitudes in patients with Parkinson’s disease that also suffered from mild cognitive impairment (MCI). A reduced P300 ERP component was also found to be associated with an increased risk for a first psychotic episode [36] (van Tricht et al., 2010). Interestingly, even criminal psychopaths show reduced P300 amplitudes [37] (Kiehl et al., 1999). Similar to our study, in all those cases, both stimuli groups elicited a P300 ERP component in both study populations (patients and controls), but there were no significant differences between ERPs elicited by high-frequency stimulus presentations (target stimuli) versus low-frequency presentations (non-target stimuli). Finally, smokers were shown to have reduced P300 ERP amplitudes in a visual oddball paradigm [38] (Anokhin et al., 2000). Clearly, reduced P300 amplitudes in the frame of an oddball paradigm seem to be a marker for various cognitive abnormalities. The fact that heavy consumers of short-video-based social media show a decreased P300 amplitude is interpreted as evidence for some sort of cognitive abnormality. At this point, the fact that we did not screen our participants for any psychological disorder that potentially could also result in reduced P300 amplitudes has to be mentioned and is seen as a limitation. However, all participants did at least self-report having no neuropathological history, no diagnosed depression, as well as no diagnosed psychological disorder.

The participant cohort of the present study included only three men in a total group of twenty-nine people, which means that the studied population was basically female. Importantly, it has been shown that different aspects of media consumption can lead to different problems related to attention and other behavioral and health-related outcomes (Cardoso-Leite et al., 2021) [19]. Regarding addiction, one interesting and relevant finding is that men seem to be more likely to lean towards internet gaming and women more likely towards social media (Su et al., 2020) [39]. In light of this finding that women are more prone to social media addiction, it might be that our finding of neurophysiologically reduced attention is more a female phenomenon than one that can be generalized. This is certainly a limitation in our study. Our participant group should have been more equally balanced between female and male participants.

A recent study found a negative relationship between school marks (GPAs) and social media use, as well as between attention and school marks. However, there was no significant interaction between social media use and attention (Barton et al., 2021) [40]. An earlier study reported about empirical evidence for a potential detrimental long-term effect of media multitasking on attention problems. However, this was only found among early adolescents but not among middle adolescents (Baumgartner et al., 2017) [41]. But, one has to emphasize that the authors of that study only collected behavioral data, requiring explicit responses from their participants mainly in the form of survey-based questions. As already highlighted in the introduction, the focus on survey-based investigations is potentially misleading. The present study introduces objective brain imaging data and shows that heavy social media users, who often watch short videos with constantly changing content demonstrate reduced attention-related brain activity. The significant finding of this study by using ERPs to describe attention-related brain activity levels might lead to an increase in using objective measures to better understand social media’s impact on humans.

## 5. Conclusions

Modern times, including digital communication and the rise of social media, have produced a drastic change in how humans deal with each other. This is all unstoppable. The possibility of reaching millions of people in less than a second is of course also tightly linked with marketing ideas and thus ways to gain money. As soon as the economy kicked in, it became clear that not only good intentions drive the further development and usage of social media. Consequently, it becomes more and more important to know as well as we can what social media do to humans. In this context, the present study was designed to focus on the impact of consuming short videos with constantly changing content on attention. Crucially, the present study utilized objective technology, which is able to watch the brain at work or, in other words, is able to ask the brain instead of asking the person carrying it around. Asking the person might lead to biased and unreliable explicit answers, whereas the brain itself always tells the truth. Via comparing calculated ERPs, the present study showed that heavy users of short-video-based social media have reduced brain activity reflecting attentional processing when compared to regular users. However, with this finding, we cannot state that there is a true causal relationship between heavy short-video-based social media consumption and reduced attention. What we found is more a correlation between heavy respective consumption and a reduced P300 ERP component. Further research is needed to find better support for a possible causal relationship. In addition, regular users should be compared with people who do not consume social media at all, and other social media content should also be focused on. Longitudinal studies should be conducted to assess the potential long-term effects, and social media consumption should be better specified. Finally, behavioral measures of attention skills should also be taken in combination with physiological measures to confirm attention deficits. However, despite these limitations, and even though the generalizability of the results of this study might be questioned due to the convenience sampling of participants, they might be useful for educational purposes and of course for the psychology discipline in general. This study is seen as a valuable and promising starting point for more research to come. The implication of highlighting attention-related problems as a result of heavy short-video-based social media consumption on brain activity levels is of great importance. Finally, our study highlights the value of completing questionnaire-based data collection with objective data collection. Thus, future studies should combine self-report data with physiological data.

## Figures and Tables

**Table 1:** ANOVA results for electrode P3. Shown are *p*-values related to condition * group interactions for each 20 ms time window separately (from 200 ms to 520 ms after stimulus onset). Due to multiple comparisons, Bonferroni correction was applied. Note that the time window from 340 to 360 ms post-stimulus onset shows the first significant interaction between the factors “condition” and “group”. This is followed by a strong trend towards significance (360–380 ms), which is followed by another significant interaction for the interval 380–400 ms and by a further strong trend towards significance (400–420 ms) (significant *p*-values are bold).

Time Window	Condition * Group		
	*p*-Value	F	Partial Eta Squared
200–220	0.144	2.259	0.077
220–240	0.183	1.865	0.065
240–260	0.686	0.167	0.006
260–280	0.945	0.005	0.000
280–300	0.550	0.367	0.013
300–320	0.602	0.278	0.010
320–340	0.165	2.041	0.070
340–360	**0.013**	7.149	0.209
360–380	0.057	3.955	0.128
380–400	**0.044**	4.454	0.142
400–420	0.081	3.292	0.109
420–440	0.122	2.554	0.086
440–460	0.317	1.037	0.037
460–480	0.065	3.710	0.121
480–500	0.189	1.814	0.063
500–520	0.172	1.968	0.068

## Data Availability

Data are available on request.
